# The Hospital Saturday Fund

**Published:** 1892-02-27

**Authors:** 


### The Hospital Saturday Fund.

The annual meeting in support of the objects of the Hospital Saturday Fund was held in the Egyptian Hall at the Mansion House, on Saturday afternoon, and was presided over by the Lord Mayor, who was supported by the Bishop of London, Sir J. Colomb, M.P., Mr. Barclay, M.P., Mr. R. B. D. Acland (Chairman of the Fund), and many of the more prominent workers in the movement, including a detachment of the St. John Ambulance Association, which has recently been associated with the Fund. The meeting was largely attended. The report of the Council for 1891 stated that they considered it a cause for congratulation that they were able to report, as the result of the year's work, that the receipts had been £19,646, as against £20,333 in 1890, and their expenses £3,103, as against £2,196 in 1890. The receipts were made up as follows: Workshop collection, £14,502; street collection, £4,912; donations, £144. The sum awarded was £15,881 15s. as against £15,682 in 1890. The number of institutions participating in the fund was 150, as against 144 in 1890. The falling off in the receipts is comparatively small when the fact is taken into account that the year has been an especially trying one to all institutions chiefly dependent upon the co-operation and support of the working classes, owing mainly to the almost continuous labour troubles, in fact the contribution of the building trade absolutely disappeared, owing to the prolonged strike. The Lord Mayor, in opening the meeting, said that the Fund, having been started in 1874, had now become a matter of everyday knowledge. It tapped a source of income that the Hospital Sunday Fund did not reach, and appealed with especial force to the working classes. The work of the hospitals was one of vital interest to all sections of the community, and did not receive the amount of public support which it deserved. (Hear, hear.) While it was true that the wealthy ought to contribute more than they did, it was equally a reflection upon the working classes that they were lax with their silver and their pence, especially as it was this class which chiefly benefited from the existence of the hospitals. Mr. Acland gave an interesting history of the Fund, dwelling especially upon the success which had attended the systematic penny-a-week shop collections established in 1889. Though, owing to labour disputes, the total amount received from this source was less than in 1890, yet the number of shops which took part in the collection had increased, and the Council hoped this increase would go on until the collection was truly representative of the whole of the working classes of this great metropolis. He defended the Fund against the charge that its establishment expenses were excessive, and paid a high tribute to the hard voluntary work of the members of the Council and the Committees. The Bishop of London moved "That this meeting views with satisfaction the increased interest taken by the working classes in the Hospital Saturday Fund." He argued that not only a section of the public, but every class was indebted to the hospitals. The Bishop observed that had it not been for the hospitals the cause of medical science would have been in a very different position to what it was to-day. On that ground alone the public should give the movement their earnest support, and in that way make the hospitals as efficient as possible. He (the Bishop) with a very warm heart moved the resolution. He deprecated the division of mankind into givers and receivers, putting in one class the humble recipients of charity, and in the other the givers of that charity. He desired that all should give in proportion to what they could, and that all should receive in proportion to their need. Upon that principle the interest taken by the working classes in this fund was based. It asserted our common brotherhood, and was in that sense alone an unmixed good. He said the collection made by workmen in their shops should be supplemented by contributions from their employers. Sir J. Colomb, M.P., seconded, and Mr. Beachcroft supported, the resolution, which was unanimously carried, as was also a further motion pledging the meeting to "use every effort to extend and consolidate" the work of the Fund. A cordial vote of thanks to the Lord Mayor for presiding concluded the proceedings.

